# Editorial: Salinity and drought stress in plants: understanding physiological, biochemical and molecular responses, volume II

**DOI:** 10.3389/fpls.2025.1625602

**Published:** 2025-06-12

**Authors:** Sunil Kumar Sahu, Pingwu Liu, Umashankar Chandrasekaran, Muhammad Waseem

**Affiliations:** ^1^ State Key Laboratory of Genome and Multi-omics Technologies, BGI Research, Shenzhen, China; ^2^ BGI Research, Wuhan, China; ^3^ School of Breeding and Multiplication (Sanya institute of Breeding and Multiplication), College of Tropical Agriculture and Forestry, Hainan University, Sanya, China; ^4^ Institute of Life Sciences, Kangwon National University, Chuncheon, Republic of Korea

**Keywords:** stress response, salinity stress, drought stress, plant physiology, molecular response, plant defense

## 1 Introduction

Salinity and drought are among the most critical abiotic stressors affecting plant growth and productivity across the globe. These challenges are particularly acute in arid and semiarid regions, where climate change has intensified water scarcity and increased soil salinization (Huang and Jin; Sahu, 2024). These stresses disrupt fundamental plant functions, including photosynthesis, nutrient uptake, and cellular homeostasis. As the global population is expected to approach 10 billion by the year 2050, agricultural systems must produce approximately 70% more food to ensure food and nutritional security (Sahu and Liu, 2023; Editorial, 2024). However, the increasing severity and frequency of salinity and drought conditions threaten this target. While severe stress might entirely limit plant development, moderate stress may activate a range of adaptive responses. These include transcriptional reprogramming, osmoprotectant buildup, alterations in stomatal behavior, and the activation of antioxidant defense systems. To create crop varieties with increased resilience, it is crucial to understand these reactions at the physiological, biochemical, and molecular levels. Recent advances in spatial omics have enabled researchers to uncover stress responses with cell-type resolution, providing new insight into how plants adapt to heterogeneous stress environments (Guo et al., 2025 ; Zhong et al., 2025). Combined with CRISPR-based gene editing, high-throughput phenotyping, bioinformatics, and integrative multiomics approaches, these tools offer powerful strategies to accelerate breeding for climate-resilient crops (Purugganan and Jackson, 2021; Wang et al., 2022; Sahu et al., 2023; Wang et al.; Zhu et al.).

**Figure 1:**
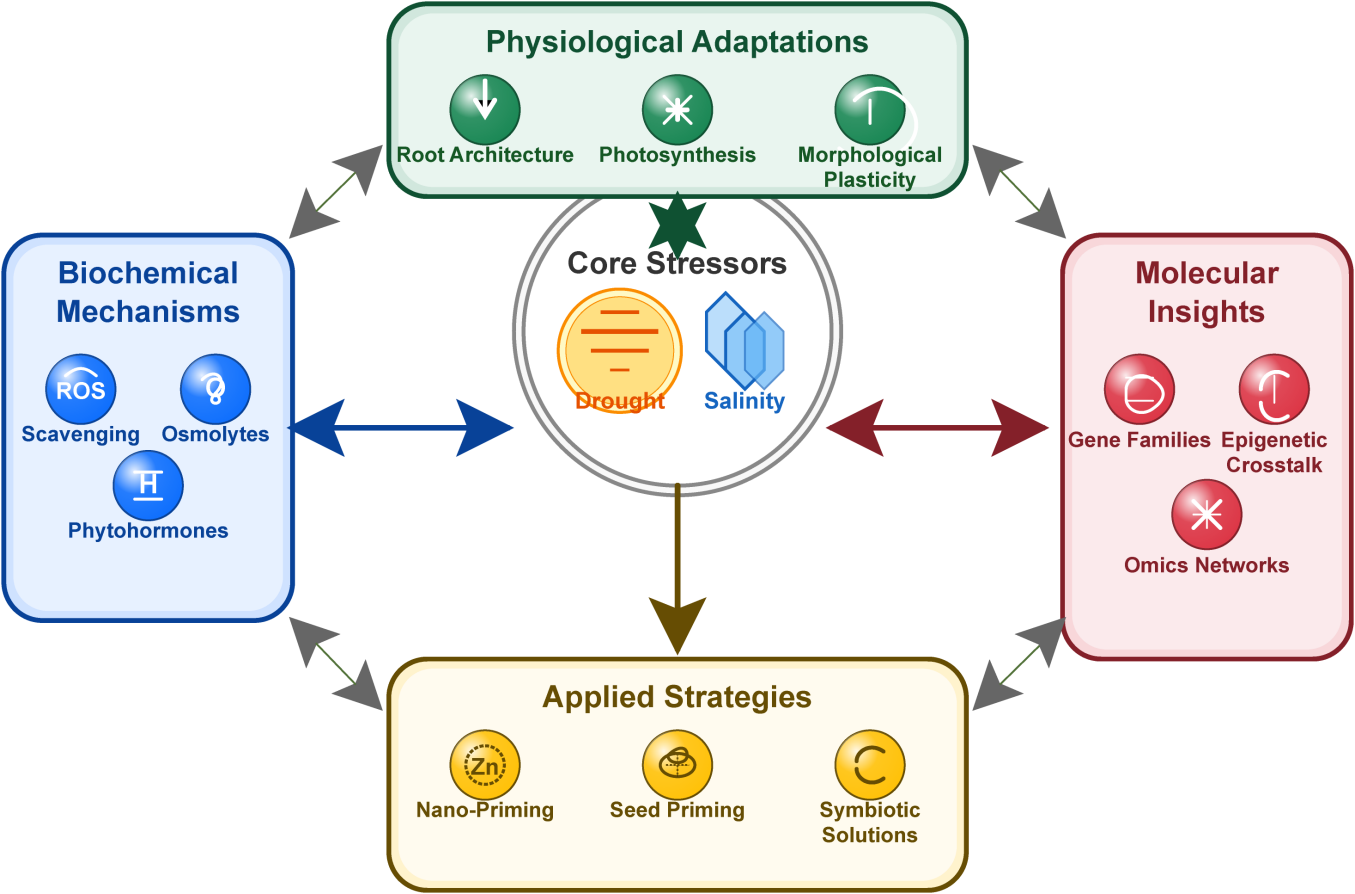
Interconnected mechanisms of plant stress adaptations to salinity and drought. The figure illustrates the conceptual framework of plant responses to salinity and drought stress, illustrating key physiological adjustments, biochemical pathways (e.g., osmolyte accumulation and antioxidant defense), and molecular networks (signal transduction and transcriptional regulation).

Volume II of “*Salinity and Drought Stress in Plants: Understanding Physiological, Biochemical and Molecular Responses*” builds on the foundation laid by the first Research Topic, focusing on integrative, translational research that bridges basic “omics” discoveries with applied breeding and biotechnology. Our Research Topic features an acceptance rate of approximately 44%, demonstrating the rigor of our peer review process and the high quality of the published contributions. The present editorial synthesizes 26 contributions comprising 23 original research articles and three review articles, organized into thematic categories that reflect the multidimensional nature of plant stress responses. It aims to contextualize the overall goals of the Research Topic, highlight advances in mechanistic insights, and provide a roadmap for future work in enhancing crop resilience (**Figure 1**).

## 2 Physiological adaptations to drought and salinity

### 2.1 Root and shoot architectural traits

Morphological plasticity underpins early stress avoidance. In chickpeas, gradual drought imposed at 70 → 50 → 25 percent water- holding capacity revealed that the MCC552 and MCC696 genotypes maintained high photosynthetic water-use efficiency and developed deeper, thicker roots—traits linked to greater proline accumulation and membrane stability (Fazeli-Nasab et al.). In oats, ear organs (glumes and lemmas) sustained water status and photosynthetic activity under drought, enabling continued carbon assimilation and grain filling even when flag leaf function declined (Fang et al.).

### 2.2 Nocturnal Water Relations and Sap Flow

The woody shrub *Cotoneaster multiflorus* utilizes nocturnal sap flow and refill mechanisms to re‐establish water balance after severe drought; stomatal closure during the day, followed by nighttime replenishment, preserves cellular hydration and supports rapid recovery upon rewatering (Huang et al.).

### 2.3 Symbiotic and nanomaterial interventions

Beneficial microbes and nanotechnology offer novel routes for physiological enhancement. In alfalfa, inoculation with *Glomus mosseae* and *G. etunicatum* improved plant height, chlorophyll content, osmolyte levels, and antioxidant enzyme activities under salt stress, underscoring the practical potential of AM fungi in saline soils (Xu et al.). Zinc oxide nanoparticles applied via nano-priming restored biomass, water content, and ion homeostasis in *Phaseolus vulgaris* under salinity, with nano-priming outperforming foliar and soil applications (Gupta et al.).

## 3 Biochemical mechanisms and metabolite regulation

### 3.1 Antioxidant defense and osmotic adjustment

The overproduction of reactive oxygen species (ROS) under stress can be mitigated through enhanced antioxidant systems and osmolyte accumulation. Tobacco seedlings treated with 0.2 mg L⁻¹ strigolactone presented elevated chlorophyll content, photosynthetic efficiency, and peroxidase/catalase activity, in addition to reduced malondialdehyde (MDA) and ROS, particularly in the moisture-sensitive cultivar Y116 (Wang et al.). In rice, seedling application of uniconazole under salt stress increased peroxidase and catalase in both tolerant (HD961) and sensitive (9311) varieties, improving root growth and carbohydrate partitioning to grains and thereby increasing yield components by up to 28 percent (Du et al.).

### 3.2 Secondary metabolites and quality prediction

Fresh leaves of *Isatis indigotica* emitted distinct biophoton signals under salt and drought, which correlated with the active ingredient levels and antibacterial efficacy of the dried herb. Delayed luminescence and spontaneous photon count serve as rapid, non-destructive indicators of cultivation quality (Wang et al.).

### 3.3 Fulvic acid and metabolomic shifts

Fulvic acid application in oats under drought increased the leaf water content and antioxidant enzyme activity, whereas integrated transcriptome–metabolome profiling revealed that the phenylpropanoid and glutathione pathways are central to FA-mediated protection (Zhu et al.).

## 4 Molecular and genomic insights

### 4.1 Gene family characterization

Recent studies have uncovered critical gene families linked to stress responses across plant species. In Moso bamboo, researchers identified 47 DIR genes, grouping them into three categories. Among these, the *PeDIR02* gene stands out— it is found in cell membranes and appears to drive rapid shoot growth while helping the plant cope with environmental stressors like drought or salinity. This gene works within a network regulated by transcription factors such as ERF, DOF, and MYB, suggesting a coordinated strategy for stress adaptation (Xuan et al.). Meanwhile, alfalfa’s nine ADF genes fall into four evolutionary subgroups. Experiments revealed that *MsADF1*, *MsADF2/3*, *MsADF6*, and *MsADF9* are strongly induced by salt and drought, indicating that actin remodeling is involved in stress adaptation (Shi et al.). Water lilies (*Nymphaea colorata*), on the other hand, boast 94 class III peroxidase genes, many of which evolved through tandem duplications. These genes activate under stressors like high salt, extreme temperatures, or heavy metals, pointing to their role in detoxification and defense (Khan et al.).

### 4.2 Functional validation in model systems

Overexpression studies have pinpointed candidate genes for crop engineering. For example, when the *AtaHMGR10* gene from Asparagus taliensis was overexpressed in Arabidopsis, the modified plants showed stronger seed germination, longer roots, and better antioxidant responses under drought, salt, and osmotic stress (Zeng et al.). Similarly, poplar trees engineered to overexpress *PagSOD2a* displayed higher antioxidant enzyme activity, lower oxidative damage (measured by reduced malondialdehyde levels), and improved growth in salty soils. The study also traced this trait’s regulation to upstream genes like SPL13, NGA1b, and FRS5 (Zhou et al.). In another experiment, a caffeic acid O-methyltransferase gene from *Ligusticum chuanxiong* boosted both lignin production and melatonin synthesis in *Arabidopsis*, enhancing drought tolerance through stronger antioxidant defenses (Huang et al.).

### 4.3 RNA-seq and population genomics

By combining RNA sequencing with population genetics, light is being shed on stress adaptation mechanisms. In flax, a merger of transcriptomics and genome-wide association studies (GWAS) pinpointed 17 salt-tolerance genes involved in pathways like phenylpropanoid biosynthesis and metal transport. Interestingly, oil flax varieties showed higher genetic diversity at these key loci compared to fiber flax, possibly explaining their greater resilience (Li et al.). For drought tolerance, transcriptome comparisons of *Codonopsis pilosula* seedlings highlighted cultivar-specific differences. The drought-resistant cultivar G1, for instance, activated unique genes tied to starch/sucrose metabolism, hormone signaling, and glutathione pathways—clues that could guide future breeding efforts (Wang et al.).

## 5 Multiomics and integrative approaches

### 5.1 Hexaploid triticale seedling responses

When salt-stressed triticale seedlings were analyzed using a mix of genome-wide association studies (GWAS), transcriptomics, and proteomics, researchers uncovered 81 genetic markers linked to stress tolerance and 688 genes/proteins that changed activity within 18 hours. A protein called LEA14 stood out as a fast-acting defender against stress, likely because its DNA contains regions targeted by multiple stress-related transcription factors (Wang et al.).

### 5.2 Dynamic salt stress regulation in maize

How do salt-tolerant and salt-sensitive maize varieties react differently to stress? A time-based study comparing the resilient SPL02 line and the sensitive Mo17 line under high salt conditions showed nearly 9,000 unique gene activity changes in each. The hardy SPL02 line relied heavily on pathways like map kinase signaling, phenylpropanoid production, and hormone regulation, while Mo17 leaned more on ABA-activated stress signaling. Five key genes, including phosphate transporters and WRKY transcription factors, were flagged as top candidates for future research (Maimaiti et al.).

## 6 Reviews and perspectives

### 6.1 Phytohormones in horticultural drought tolerance

A comprehensive review highlights the exogenous application of melatonin, salicylic acid, jasmonates, strigolactones, brassinosteroids, and γ-aminobutyric acid as modulators of molecular and physiological defense systems in horticultural crops under drought, emphasizing hormone crosstalk and integrated signaling networks. The authors stress the need to decode these complex signaling networks to improve crop resilience (Huang and Jin).

### 6.2 Fibrillins and redox signaling in response to multi-stress

Two studies shed light on stress adaptation strategies. One focuses on fibrillins, proteins in plant plastids that help manage lipid storage and oxidative stress. The other proposes a new model where epigenetic changes (like DNA methylation) and redox signaling work together to fine-tune plant responses to combined stressors. Both papers argue for targeting these mechanisms in breeding programs to create tougher crops (El-Sappah et al.; Shriti et al).

## 7 Concluding remarks

The research presented in this Research Topic showcases how blending insights from genetics, physiology, and cutting-edge technology can unlock the secrets of plant resilience. By studying everything from root and shoot structures to antioxidant systems and gene networks, scientists are uncovering how plants survive droughts and salty soils. The real challenge lies in transforming these discoveries into practical solutions. To succeed, researchers, breeders, and farmers will need to collaborate closely, harnessing tools like gene editing, precision breeding, and smarter agricultural practices. Together, these efforts could pave the way for crops that thrive in our planet’s increasingly harsh environments, helping protect food supplies and livelihoods for future generations.
